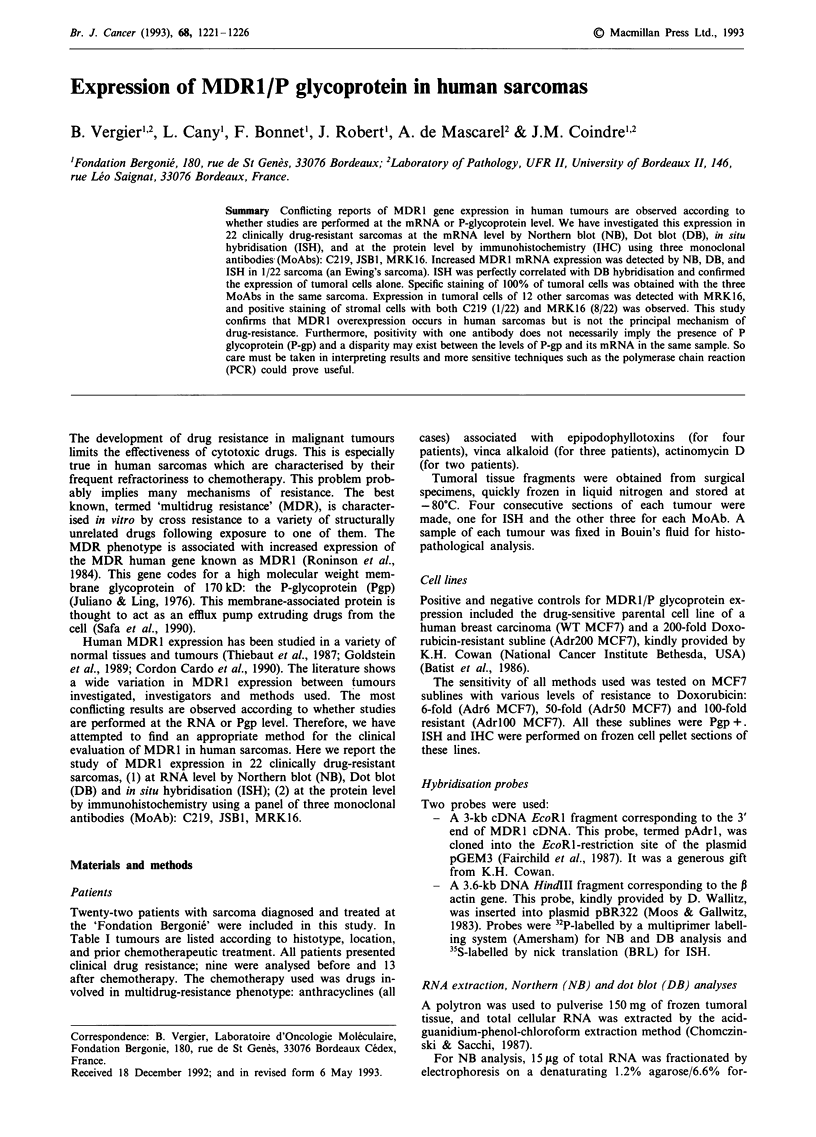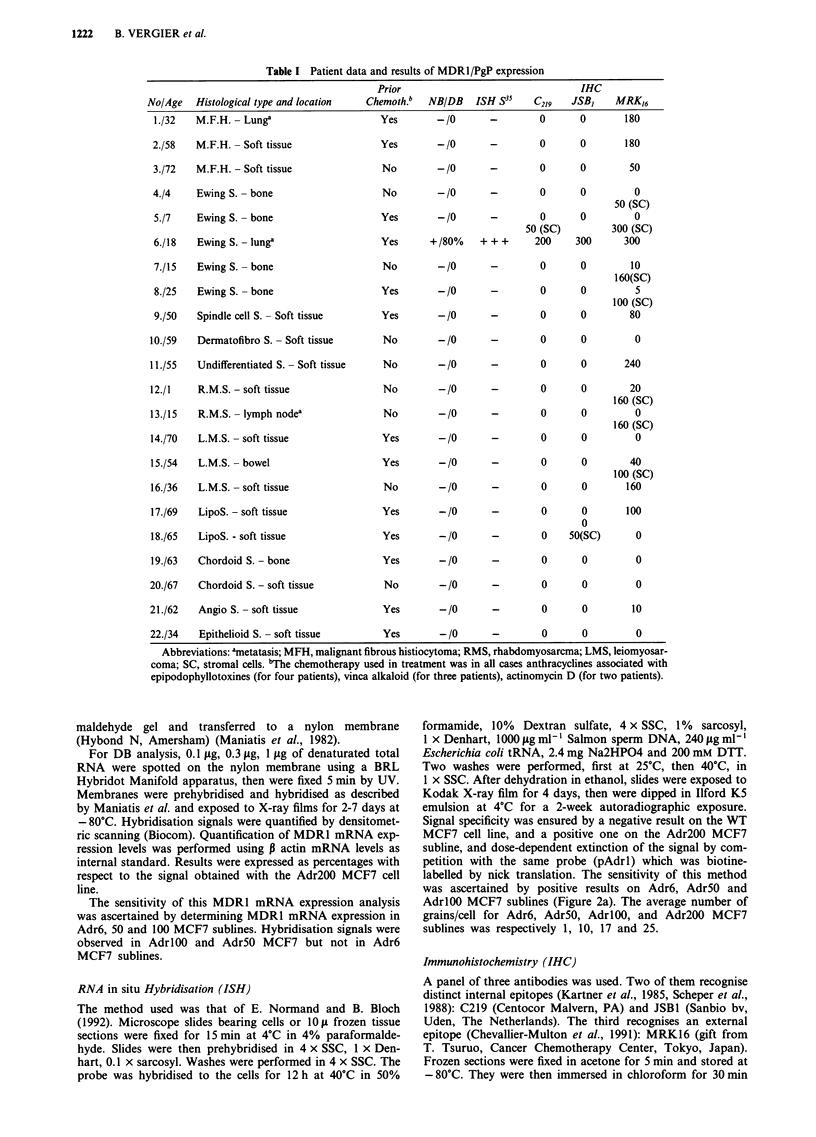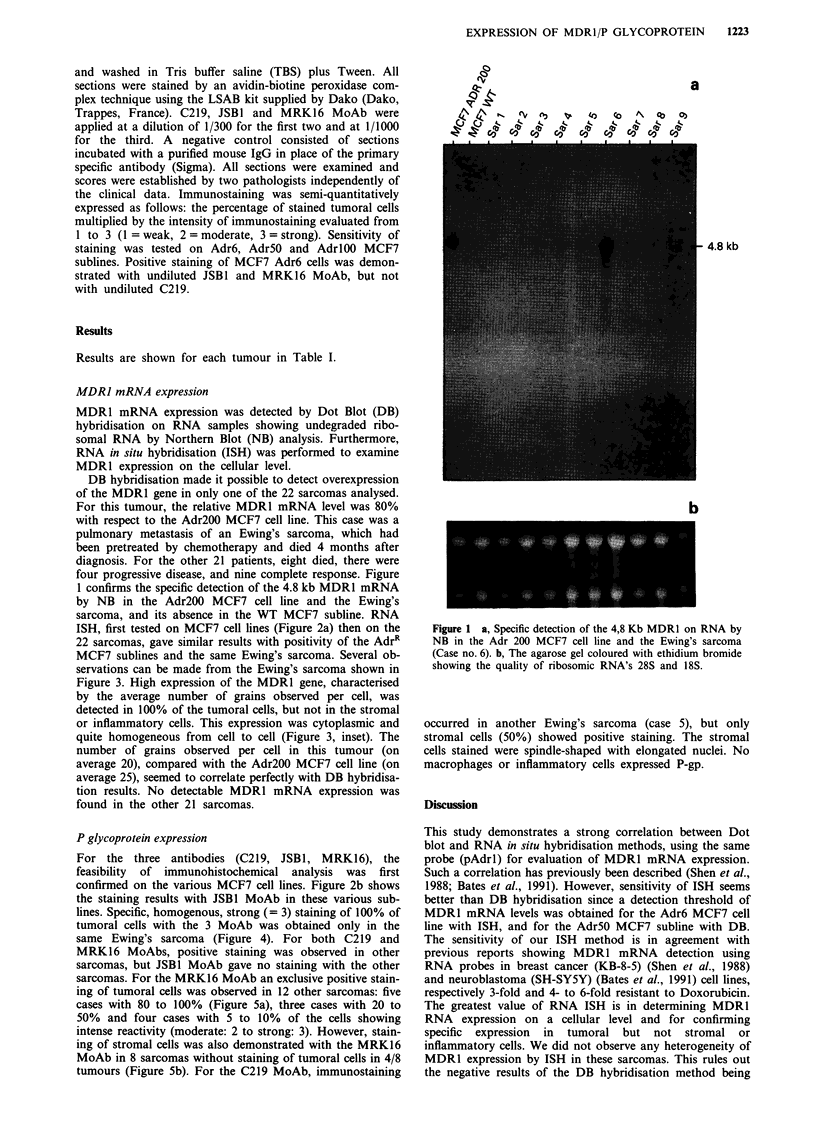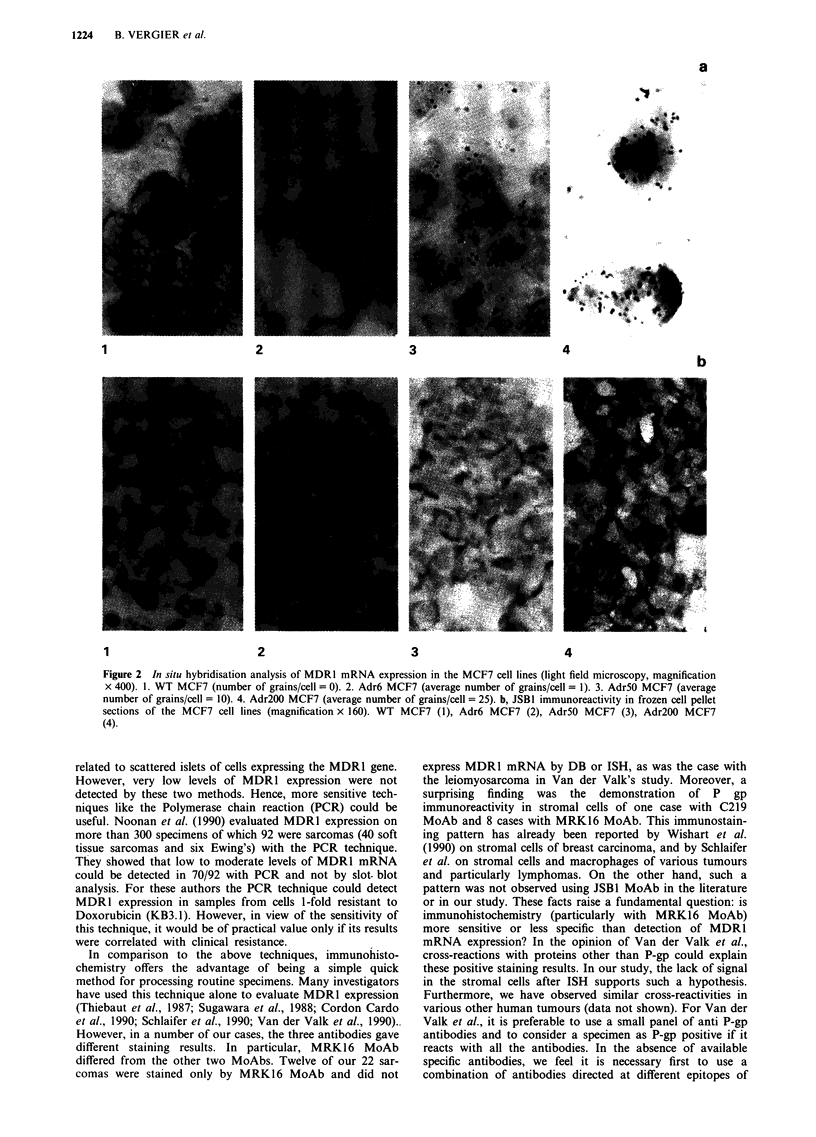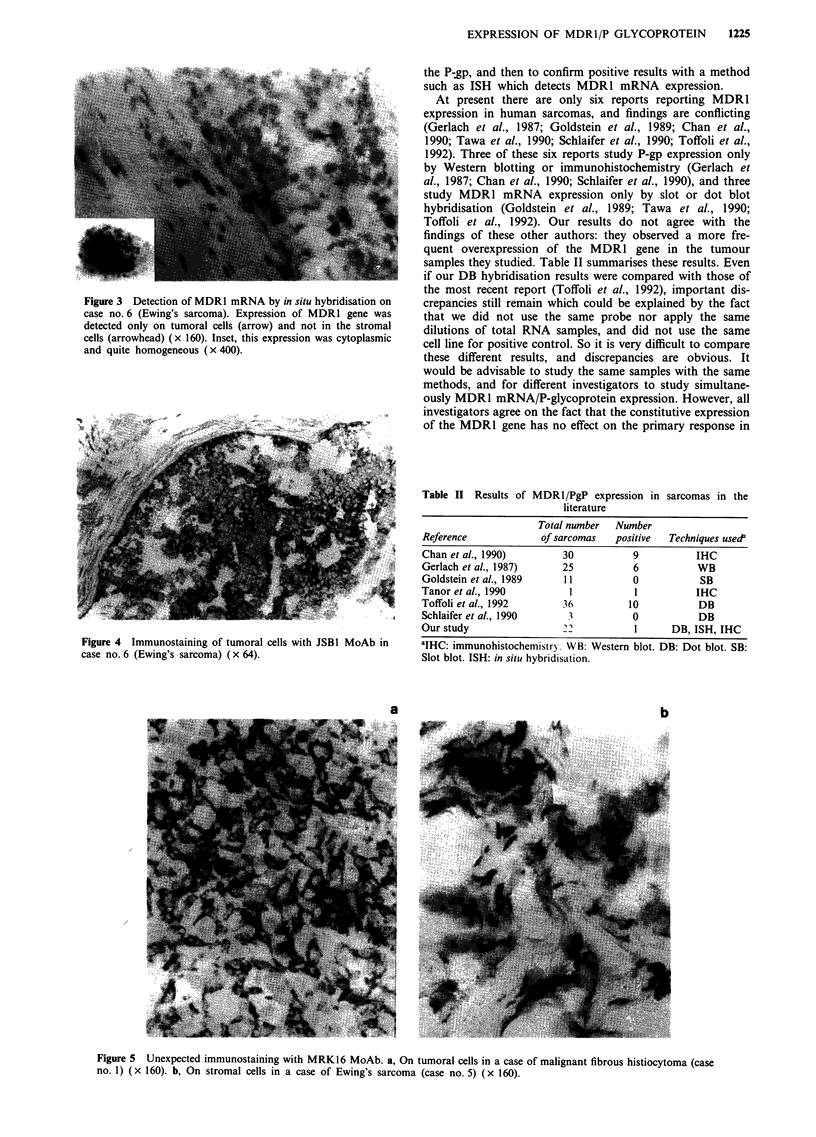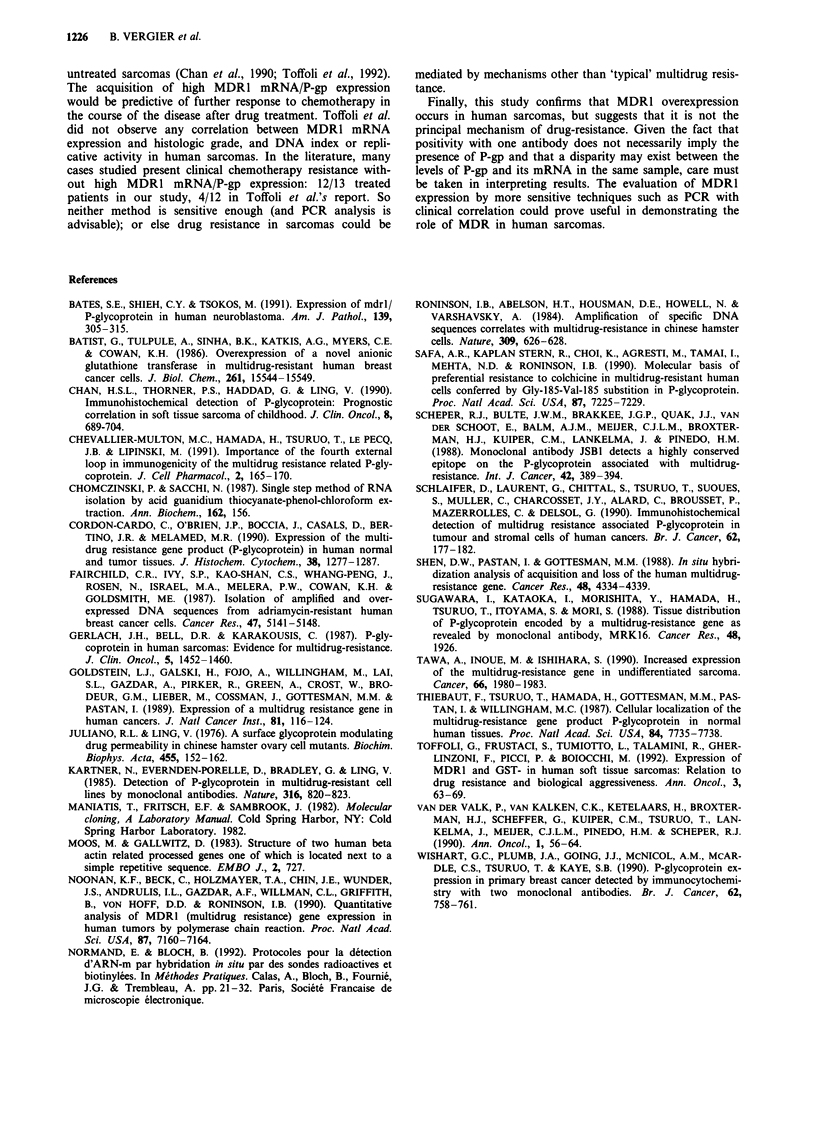# Expression of MDR1/P glycoprotein in human sarcomas.

**DOI:** 10.1038/bjc.1993.508

**Published:** 1993-12

**Authors:** B. Vergier, L. Cany, F. Bonnet, J. Robert, A. de Mascarel, J. M. Coindre

**Affiliations:** Fondation Bergonié, Bordeaux, France.

## Abstract

**Images:**


Br. J. Cancer (1993), 68, 1221–1226 © Macmillan Press Ltd., 1993

### Expression of MDR1/P glycoprotein in human sarcomas

B. Vergier[1,2], L. Cany[1], F. Bonnet[1], J. Robert[1], A. de Mascarel[2] & J.M. Coindre[1,2]

[1]*Fondation Bergonié, 180, rue de St Genès, 33076 Bordeaux;* [2]*Laboratory of Pathology, UFR II, University of Bordeaux II, 146, rue Léo Saignat, 33076 Bordeaux, France.*

**Summary** Conflicting reports of MDR1 gene expression in human tumours are observed according to whether studies are performed at the mRNA or P-glycoprotein level. We have investigated this expression in 22 clinically drug-resistant sarcomas at the mRNA level by Northern blot (NB), Dot blot (DB), *in situ* hybridisation (ISH), and at the protein level by immunohistochemistry (IHC) using three monoclonal antibodies (MoAbs): C219, JSB1, MRK16. Increased MDR1 mRNA expression was detected by NB, DB, and ISH in 1/22 sarcoma (an Ewing's sarcoma). ISH was perfectly correlated with DB hybridisation and confirmed the expression of tumoral cells alone. Specific staining of 100% of tumoral cells was obtained with the three MoAbs in the same sarcoma. Expression in tumoral cells of 12 other sarcomas was detected with MRK16, and positive staining of stromal cells with both C219 (1/22) and MRK16 (8/22) was observed. This study confirms that MDR1 overexpression occurs in human sarcomas but is not the principal mechanism of drug-resistance. Furthermore, positivity with one antibody does not necessarily imply the presence of P glycoprotein (P-gp) and a disparity may exist between the levels of P-gp and its mRNA in the same sample. So care must be taken in interpreting results and more sensitive techniques such as the polymerase chain reaction (PCR) could prove useful.

The development of drug resistance in malignant tumours limits the effectiveness of cytotoxic drugs. This is especially true in human sarcomas which are characterised by their frequent refractoriness to chemotherapy. This problem probably implies many mechanisms of resistance. The best known, termed 'multidrug resistance' (MDR), is characterised *in vitro* by cross resistance to a variety of structurally unrelated drugs following exposure to one of them. The MDR phenotype is associated with increased expression of the MDR human gene known as MDR1 (Roninson *et al.*, 1984). This gene codes for a high molecular weight membrane glycoprotein of 170 kD: the P-glycoprotein (Pgp) (Juliano & Ling, 1976). This membrane-associated protein is thought to act as an efflux pump extruding drugs from the cell (Safa *et al.*, 1990).

Human MDR1 expression has been studied in a variety of normal tissues and tumours (Thiebaut *et al.*, 1987; Goldstein *et al.*, 1989; Cordon Cardo *et al.*, 1990). The literature shows a wide variation in MDR1 expression between tumours investigated, investigators and methods used. The most conflicting results are observed according to whether studies are performed at the RNA or Pgp level. Therefore, we have attempted to find an appropriate method for the clinical evaluation of MDR1 in human sarcomas. Here we report the study of MDR1 expression in 22 clinically drug-resistant sarcomas, (1) at RNA level by Northern blot (NB), Dot blot (DB) and *in situ* hybridisation (ISH); (2) at the protein level by immunohistochemistry using a panel of three monoclonal antibodies (MoAb): C219, JSB1, MRK16.

### Materials and methods

### *Patients*

Twenty-two patients with sarcoma diagnosed and treated at the 'Fondation Bergonié' were included in this study. In Table I tumours are listed according to histotype, location, and prior chemotherapeutic treatment. All patients presented clinical drug resistance; nine were analysed before and 13 after chemotherapy. The chemotherapy used was drugs involved in multidrug-resistance phenotype: anthracyclines (all cases) associated with epipodophyllotoxins (for four patients), vinca alkaloid (for three patients), actinomycin D (for two patients).

Tumoral tissue fragments were obtained from surgical specimens, quickly frozen in liquid nitrogen and stored at −80°C. Four consecutive sections of each tumour were made, one for ISH and the other three for each MoAb. A sample of each tumour was fixed in Bouin's fluid for histopathological analysis.

### *Cell lines*

Positive and negative controls for MDR1/P glycoprotein expression included the drug-sensitive parental cell line of a human breast carcinoma (WT MCF7) and a 200-fold Doxorubicin-resistant subline (Adr200 MCF7), kindly provided by K.H. Cowan (National Cancer Institute Bethesda, USA) (Batist *et al.*, 1986).

The sensitivity of all methods used was tested on MCF7 sublines with various levels of resistance to Doxorubicin: 6-fold (Adr6 MCF7), 50-fold (Adr50 MCF7) and 100-fold resistant (Adr100 MCF7). All these sublines were Pgp +. ISH and IHC were performed on frozen cell pellet sections of these lines.

### *Hybridisation probes*

Two probes were used:

- A 3-kb cDNA *Eco*R1 fragment corresponding to the 3′ end of MDR1 cDNA. This probe, termed pAdr1, was cloned into the *Eco*R1-restriction site of the plasmid pGEM3 (Fairchild *et al.*, 1987). It was a generous gift from K.H. Cowan.
- A 3.6-kb DNA *Hind*III fragment corresponding to the β actin gene. This probe, kindly provided by D. Wallitz, was inserted into plasmid pBR322 (Moos & Gallwitz, 1983). Probes were $^{32}$P-labelled by a multiprimer labelling system (Amersham) for NB and DB analysis and $^{35}$S-labelled by nick translation (BRL) for ISH.

### *RNA extraction, Northern (NB) and dot blot (DB) analyses*

A polytron was used to pulverise 150 mg of frozen tumoral tissue, and total cellular RNA was extracted by the acid-guanidium-phenol-chloroform extraction method (Chomczinski & Sacchi, 1987).

For NB analysis, 15 μg of total RNA was fractionated by electrophoresis on a denaturating 1.2% agarose/6.6% for-

Correspondence: B. Vergier, Laboratoire d'Oncologie Moléculaire, Fondation Bergonie, 180, rue de St Genès, 33076 Bordeaux Cédex, France.
Received 18 December 1992; and in revised form 6 May 1993.

**Table I** Patient data and results of MDR1/PgP expression

| No/Age | Histological type and location | Prior Chemoth.[b] | NB/DB | ISH $S^{35}$ | IHC $C_{219}$ | $JSB_1$ | $MRK_{16}$ |
|---|---|---|---|---|---|---|---|
| 1./32 | M.F.H. - Lung[a] | Yes | –/0 | – | 0 | 0 | 180 |
| 2./58 | M.F.H. - Soft tissue | Yes | –/0 | – | 0 | 0 | 180 |
| 3./72 | M.F.H. - Soft tissue | No | –/0 | – | 0 | 0 | 50 |
| 4./4 | Ewing S. - bone | No | –/0 | – | 0 | 0 | 0 |
| 5./7 | Ewing S. - bone | Yes | –/0 | – | 0 | 0 | 50 (SC)<br>0 |
| 6./18 | Ewing S. - lung[a] | Yes | +/80% | + + + | 50 (SC)<br>200 | 300 | 300 (SC)<br>300 |
| 7./15 | Ewing S. - bone | No | –/0 | – | 0 | 0 | 10 |
| 8./25 | Ewing S. - bone | Yes | –/0 | – | 0 | 0 | 160(SC)<br>5 |
| 9./50 | Spindle cell S. - Soft tissue | Yes | –/0 | – | 0 | 0 | 100 (SC)<br>80 |
| 10./59 | Dermatofibro S. - Soft tissue | No | –/0 | – | 0 | 0 | 0 |
| 11./55 | Undifferentiated S. - Soft tissue | No | –/0 | – | 0 | 0 | 240 |
| 12./1 | R.M.S. - soft tissue | No | –/0 | – | 0 | 0 | 20 |
| 13./15 | R.M.S. - lymph node[a] | No | –/0 | – | 0 | 0 | 160 (SC)<br>0 |
| 14./70 | L.M.S. - soft tissue | Yes | –/0 | – | 0 | 0 | 160 (SC)<br>0 |
| 15./54 | L.M.S. - bowel | Yes | –/0 | – | 0 | 0 | 40 |
| 16./36 | L.M.S. - soft tissue | No | –/0 | – | 0 | 0 | 100 (SC)<br>160 |
| 17./69 | LipoS. - soft tissue | Yes | –/0 | – | 0 | 0 | 100 |
| 18./65 | LipoS. - soft tissue | Yes | –/0 | – | 0 | 0<br>50(SC) | 0 |
| 19./63 | Chordoid S. - bone | Yes | –/0 | – | 0 | 0 | 0 |
| 20./67 | Chordoid S. - soft tissue | No | –/0 | – | 0 | 0 | 0 |
| 21./62 | Angio S. - soft tissue | Yes | –/0 | – | 0 | 0 | 10 |
| 22./34 | Epithelioid S. - soft tissue | Yes | –/0 | – | 0 | 0 | 0 |

Abbreviations: [a]metatasis; MFH, malignant fibrous histiocytoma; RMS, rhabdomyosarcma; LMS, leiomyosarcoma; SC, stromal cells. [b]The chemotherapy used in treatment was in all cases anthracyclines associated with epipodophyllotoxines (for four patients), vinca alkaloid (for three patients), actinomycin D (for two patients).

maldehyde gel and transferred to a nylon membrane (Hybond N, Amersham) (Maniatis et al., 1982).

For DB analysis, 0.1 μg, 0.3 μg, 1 μg of denaturated total RNA were spotted on the nylon membrane using a BRL Hybridot Manifold apparatus, then were fixed 5 min by UV. Membranes were prehybridised and hybridised as described by Maniatis et al. and exposed to X-ray films for 2-7 days at −80°C. Hybridisation signals were quantified by densitometric scanning (Biocom). Quantification of MDR1 mRNA expression levels was performed using β actin mRNA levels as internal standard. Results were expressed as percentages with respect to the signal obtained with the Adr200 MCF7 cell line.

The sensitivity of this MDR1 mRNA expression analysis was ascertained by determining MDR1 mRNA expression in Adr6, 50 and 100 MCF7 sublines. Hybridisation signals were observed in Adr100 and Adr50 MCF7 but not in Adr6 MCF7 sublines.

### *RNA* in situ *Hybridisation (ISH)*

The method used was that of E. Normand and B. Bloch (1992). Microscope slides bearing cells or 10 μ frozen tissue sections were fixed for 15 min at 4°C in 4% paraformaldehyde. Slides were then prehybridised in 4 × SSC, 1 × Denhart, 0.1 × sarcosyl. Washes were performed in 4 × SSC. The probe was hybridised to the cells for 12 h at 40°C in 50% formamide, 10% Dextran sulfate, 4 × SSC, 1% sarcosyl, 1 × Denhart, 1000 μg $ml^{-1}$ Salmon sperm DNA, 240 μg $ml^{-1}$ *Escherichia coli* tRNA, 2.4 mg Na2HPO4 and 200 mM DTT. Two washes were performed, first at 25°C, then 40°C, in 1 × SSC. After dehydration in ethanol, slides were exposed to Kodak X-ray film for 4 days, then were dipped in Ilford K5 emulsion at 4°C for a 2-week autoradiographic exposure. Signal specificity was ensured by a negative result on the WT MCF7 cell line, and a positive one on the Adr200 MCF7 subline, and dose-dependent extinction of the signal by competition with the same probe (pAdr1) which was biotine-labelled by nick translation. The sensitivity of this method was ascertained by positive results on Adr6, Adr50 and Adr100 MCF7 sublines (Figure 2a). The average number of grains/cell for Adr6, Adr50, Adr100, and Adr200 MCF7 sublines was respectively 1, 10, 17 and 25.

### *Immunohistochemistry (IHC)*

A panel of three antibodies was used. Two of them recognise distinct internal epitopes (Kartner et al., 1985, Scheper et al., 1988): C219 (Centocor Malvern, PA) and JSB1 (Sanbio bv, Uden, The Netherlands). The third recognises an external epitope (Chevallier-Multon et al., 1991): MRK16 (gift from T. Tsuruo, Cancer Chemotherapy Center, Tokyo, Japan). Frozen sections were fixed in acetone for 5 min and stored at −80°C. They were then immersed in chloroform for 30 min and washed in Tris buffer saline (TBS) plus Tween. All sections were stained by an avidin-biotine peroxidase complex technique using the LSAB kit supplied by Dako (Dako, Trappes, France). C219, JSB1 and MRK16 MoAb were applied at a dilution of 1/300 for the first two and at 1/1000 for the third. A negative control consisted of sections incubated with a purified mouse IgG in place of the primary specific antibody (Sigma). All sections were examined and scores were established by two pathologists independently of the clinical data. Immunostaining was semi-quantitatively expressed as follows: the percentage of stained tumoral cells multiplied by the intensity of immunostaining evaluated from 1 to 3 (1 = weak, 2 = moderate, 3 = strong). Sensitivity of staining was tested on Adr6, Adr50 and Adr100 MCF7 sublines. Positive staining of MCF7 Adr6 cells was demonstrated with undiluted JSB1 and MRK16 MoAb, but not with undiluted C219.

### Results

Results are shown for each tumour in Table I.

### *MDR1 mRNA expression*

MDR1 mRNA expression was detected by Dot Blot (DB) hybridisation on RNA samples showing undegraded ribosomal RNA by Northern Blot (NB) analysis. Furthermore, RNA *in situ* hybridisation (ISH) was performed to examine MDR1 expression on the cellular level.

DB hybridisation made it possible to detect overexpression of the MDR1 gene in only one of the 22 sarcomas analysed. For this tumour, the relative MDR1 mRNA level was 80% with respect to the Adr200 MCF7 cell line. This case was a pulmonary metastasis of an Ewing's sarcoma, which had been pretreated by chemotherapy and died 4 months after diagnosis. For the other 21 patients, eight died, there were four progressive disease, and nine complete response. Figure 1 confirms the specific detection of the 4.8 kb MDR1 mRNA by NB in the Adr200 MCF7 cell line and the Ewing's sarcoma, and its absence in the WT MCF7 subline. RNA ISH, first tested on MCF7 cell lines (Figure 2a) then on the 22 sarcomas, gave similar results with positivity of the Adr$^R$ MCF7 sublines and the same Ewing's sarcoma. Several observations can be made from the Ewing's sarcoma shown in Figure 3. High expression of the MDR1 gene, characterised by the average number of grains observed per cell, was detected in 100% of the tumoral cells, but not in the stromal or inflammatory cells. This expression was cytoplasmic and quite homogeneous from cell to cell (Figure 3, inset). The number of grains observed per cell in this tumour (on average 20), compared with the Adr200 MCF7 cell line (on average 25), seemed to correlate perfectly with DB hybridisation results. No detectable MDR1 mRNA expression was found in the other 21 sarcomas.

### *P glycoprotein expression*

For the three antibodies (C219, JSB1, MRK16), the feasibility of immunohistochemical analysis was first confirmed on the various MCF7 cell lines. Figure 2b shows the staining results with JSB1 MoAb in these various sublines. Specific, homogenous, strong (= 3) staining of 100% of tumoral cells with the 3 MoAb was obtained only in the same Ewing's sarcoma (Figure 4). For both C219 and MRK16 MoAbs, positive staining was observed in other sarcomas, but JSB1 MoAb gave no staining with the other sarcomas. For the MRK16 MoAb an exclusive positive staining of tumoral cells was observed in 12 other sarcomas: five cases with 80 to 100% (Figure 5a), three cases with 20 to 50% and four cases with 5 to 10% of the cells showing intense reactivity (moderate: 2 to strong: 3). However, staining of stromal cells was also demonstrated with the MRK16 MoAb in 8 sarcomas without staining of tumoral cells in 4/8 tumours (Figure 5b). For the C219 MoAb, immunostaining

**Figure 1** **a**, Specific detection of the 4,8 Kb MDR1 on RNA by NB in the Adr 200 MCF7 cell line and the Ewing's sarcoma (Case no. 6). **b**, The agarose gel coloured with ethidium bromide showing the quality of ribosomic RNA's 28S and 18S.

occurred in another Ewing's sarcoma (case 5), but only stromal cells (50%) showed positive staining. The stromal cells stained were spindle-shaped with elongated nuclei. No macrophages or inflammatory cells expressed P-gp.

### Discussion

This study demonstrates a strong correlation between Dot blot and RNA *in situ* hybridisation methods, using the same probe (pAdr1) for evaluation of MDR1 mRNA expression. Such a correlation has previously been described (Shen *et al.*, 1988; Bates *et al.*, 1991). However, sensitivity of ISH seems better than DB hybridisation since a detection threshold of MDR1 mRNA levels was obtained for the Adr6 MCF7 cell line with ISH, and for the Adr50 MCF7 subline with DB. The sensitivity of our ISH method is in agreement with previous reports showing MDR1 mRNA detection using RNA probes in breast cancer (KB-8-5) (Shen *et al.*, 1988) and neuroblastoma (SH-SY5Y) (Bates *et al.*, 1991) cell lines, respectively 3-fold and 4- to 6-fold resistant to Doxorubicin. The greatest value of RNA ISH is in determining MDR1 RNA expression on a cellular level and for confirming specific expression in tumoral but not stromal or inflammatory cells. We did not observe any heterogeneity of MDR1 expression by ISH in these sarcomas. This rules out the negative results of the DB hybridisation method being

**Figure 2** *In situ* hybridisation analysis of MDR1 mRNA expression in the MCF7 cell lines (light field microscopy, magnification × 400). 1. WT MCF7 (number of grains/cell = 0). 2. Adr6 MCF7 (average number of grains/cell = 1). 3. Adr50 MCF7 (average number of grains/cell = 10). 4. Adr200 MCF7 (average number of grains/cell = 25). **b**, JSB1 immunoreactivity in frozen cell pellet sections of the MCF7 cell lines (magnification × 160). WT MCF7 (1), Adr6 MCF7 (2), Adr50 MCF7 (3), Adr200 MCF7 (4).

related to scattered islets of cells expressing the MDR1 gene. However, very low levels of MDR1 expression were not detected by these two methods. Hence, more sensitive techniques like the Polymerase chain reaction (PCR) could be useful. Noonan *et al.* (1990) evaluated MDR1 expression on more than 300 specimens of which 92 were sarcomas (40 soft tissue sarcomas and six Ewing's) with the PCR technique. They showed that low to moderate levels of MDR1 mRNA could be detected in 70/92 with PCR and not by slot- blot analysis. For these authors the PCR technique could detect MDR1 expression in samples from cells 1-fold resistant to Doxorubicin (KB3.1). However, in view of the sensitivity of this technique, it would be of practical value only if its results were correlated with clinical resistance.

In comparison to the above techniques, immunohistochemistry offers the advantage of being a simple quick method for processing routine specimens. Many investigators have used this technique alone to evaluate MDR1 expression (Thiebaut *et al.*, 1987; Sugawara *et al.*, 1988; Cordon Cardo *et al.*, 1990; Schlaifer *et al.*, 1990; Van der Valk *et al.*, 1990). However, in a number of our cases, the three antibodies gave different staining results. In particular, MRK16 MoAb differed from the other two MoAbs. Twelve of our 22 sarcomas were stained only by MRK16 MoAb and did not express MDR1 mRNA by DB or ISH, as was the case with the leiomyosarcoma in Van der Valk's study. Moreover, a surprising finding was the demonstration of P gp immunoreactivity in stromal cells of one case with C219 MoAb and 8 cases with MRK16 MoAb. This immunostaining pattern has already been reported by Wishart *et al.* (1990) on stromal cells of breast carcinoma, and by Schlaifer *et al.* on stromal cells and macrophages of various tumours and particularly lymphomas. On the other hand, such a pattern was not observed using JSB1 MoAb in the literature or in our study. These facts raise a fundamental question: is immunohistochemistry (particularly with MRK16 MoAb) more sensitive or less specific than detection of MDR1 mRNA expression? In the opinion of Van der Valk *et al.*, cross-reactions with proteins other than P-gp could explain these positive staining results. In our study, the lack of signal in the stromal cells after ISH supports such a hypothesis. Furthermore, we have observed similar cross-reactivities in various other human tumours (data not shown). For Van der Valk *et al.*, it is preferable to use a small panel of anti P-gp antibodies and to consider a specimen as P-gp positive if it reacts with all the antibodies. In the absence of available specific antibodies, we feel it is necessary first to use a combination of antibodies directed at different epitopes of

**Figure 3** Detection of MDR1 mRNA by *in situ* hybridisation on case no. 6 (Ewing's sarcoma). Expression of MDR1 gene was detected only on tumoral cells (arrow) and not in the stromal cells (arrowhead) (× 160). Inset, this expression was cytoplasmic and quite homogeneous (× 400).

**Figure 4** Immunostaining of tumoral cells with JSB1 MoAb in case no. 6 (Ewing's sarcoma) (× 64).

the P-gp, and then to confirm positive results with a method such as ISH which detects MDR1 mRNA expression.

At present there are only six reports reporting MDR1 expression in human sarcomas, and findings are conflicting (Gerlach *et al.*, 1987; Goldstein *et al.*, 1989; Chan *et al.*, 1990; Tawa *et al.*, 1990; Schlaifer *et al.*, 1990; Toffoli *et al.*, 1992). Three of these six reports study P-gp expression only by Western blotting or immunohistochemistry (Gerlach *et al.*, 1987; Chan *et al.*, 1990; Schlaifer *et al.*, 1990), and three study MDR1 mRNA expression only by slot or dot blot hybridisation (Goldstein *et al.*, 1989; Tawa *et al.*, 1990; Toffoli *et al.*, 1992). Our results do not agree with the findings of these other authors: they observed a more frequent overexpression of the MDR1 gene in the tumour samples they studied. Table II summarises these results. Even if our DB hybridisation results were compared with those of the most recent report (Toffoli *et al.*, 1992), important discrepancies still remain which could be explained by the fact that we did not use the same probe nor apply the same dilutions of total RNA samples, and did not use the same cell line for positive control. So it is very difficult to compare these different results, and discrepancies are obvious. It would be advisable to study the same samples with the same methods, and for different investigators to study simultaneously MDR1 mRNA/P-glycoprotein expression. However, all investigators agree on the fact that the constitutive expression of the MDR1 gene has no effect on the primary response in

**Table II** Results of MDR1/PgP expression in sarcomas in the literature

| *Reference* | *Total number of sarcomas* | *Number positive* | *Techniques used*[a] |
|---|---|---|---|
| Chan *et al.*, 1990) | 30 | 9 | IHC |
| Gerlach *et al.*, 1987) | 25 | 6 | WB |
| Goldstein *et al.*, 1989 | 11 | 0 | SB |
| Tanor *et al.*, 1990 | 1 | 1 | IHC |
| Toffoli *et al.*, 1992 | 36 | 10 | DB |
| Schlaifer *et al.*, 1990 | 3 | 0 | DB |
| Our study | 22 | 1 | DB, ISH, IHC |

[a]IHC: immunohistochemistry. WB: Western blot. DB: Dot blot. SB: Slot blot. ISH: *in situ* hybridisation.

**Figure 5** Unexpected immunostaining with MRK16 MoAb. **a**, On tumoral cells in a case of malignant fibrous histiocytoma (case no. 1) (× 160). **b**, On stromal cells in a case of Ewing's sarcoma (case no. 5) (× 160).

untreated sarcomas (Chan et al., 1990; Toffoli et al., 1992). The acquisition of high MDR1 mRNA/P-gp expression would be predictive of further response to chemotherapy in the course of the disease after drug treatment. Toffoli et al. did not observe any correlation between MDR1 mRNA expression and histologic grade, and DNA index or replicative activity in human sarcomas. In the literature, many cases studied present clinical chemotherapy resistance without high MDR1 mRNA/P-gp expression: 12/13 treated patients in our study, 4/12 in Toffoli et al.'s report. So neither method is sensitive enough (and PCR analysis is advisable); or else drug resistance in sarcomas could be mediated by mechanisms other than 'typical' multidrug resistance.

Finally, this study confirms that MDR1 overexpression occurs in human sarcomas, but suggests that it is not the principal mechanism of drug-resistance. Given the fact that positivity with one antibody does not necessarily imply the presence of P-gp and that a disparity may exist between the levels of P-gp and its mRNA in the same sample, care must be taken in interpreting results. The evaluation of MDR1 expression by more sensitive techniques such as PCR with clinical correlation could prove useful in demonstrating the role of MDR in human sarcomas.

### References

BATES, S.E., SHIEH, C.Y. & TSOKOS, M. (1991). Expression of mdr1/P-glycoprotein in human neuroblastoma. *Am. J. Pathol.*, **139**, 305–315.

BATIST, G., TULPULE, A., SINHA, B.K., KATKIS, A.G., MYERS, C.E. & COWAN, K.H. (1986). Overexpression of a novel anionic glutathione transferase in multidrug-resistant human breast cancer cells. *J. Biol. Chem.*, **261**, 15544–15549.

CHAN, H.S.L., THORNER, P.S., HADDAD, G. & LING, V. (1990). Immunohistochemical detection of P-glycoprotein: Prognostic correlation in soft tissue sarcoma of childhood. *J. Clin. Oncol.*, **8**, 689-704.

CHEVALLIER-MULTON, M.C., HAMADA, H., TSURUO, T., LE PECQ, J.B. & LIPINSKI, M. (1991). Importance of the fourth external loop in immunogenicity of the multidrug resistance related P-glycoprotein. *J. Cell Pharmacol.*, **2**, 165–170.

CHOMCZINSKI, P. & SACCHI, N. (1987). Single step method of RNA isolation by acid guanidium thiocyanate-phenol-chloroform extraction. *Ann. Biochem.*, **162**, 156.

CORDON-CARDO, C., O'BRIEN, J.P., BOCCIA, J., CASALS, D., BERTINO, J.R. & MELAMED, M.R. (1990). Expression of the multidrug resistance gene product (P-glycoprotein) in human normal and tumor tissues. *J. Histochem. Cytochem.*, **38**, 1277–1287.

FAIRCHILD, C.R., IVY, S.P., KAO-SHAN, C.S., WHANG-PENG, J., ROSEN, N., ISRAEL, M.A., MELERA, P.W., COWAN, K.H. & GOLDSMITH, ME. (1987). Isolation of amplified and overexpressed DNA sequences from adriamycin-resistant human breast cancer cells. *Cancer Res.*, **47**, 5141–5148.

GERLACH, J.H., BELL, D.R. & KARAKOUSIS, C. (1987). P-glycoprotein in human sarcomas: Evidence for multidrug-resistance. *J. Clin. Oncol.*, **5**, 1452–1460.

GOLDSTEIN, L.J., GALSKI, H., FOJO, A., WILLINGHAM, M., LAI, S.L., GAZDAR, A., PIRKER, R., GREEN, A., CROST, W., BRODEUR, G.M., LIEBER, M., COSSMAN, J., GOTTESMAN, M.M. & PASTAN, I. (1989). Expression of a multidrug resistance gene in human cancers. *J. Natl Cancer Inst.*, **81**, 116–124.

JULIANO, R.L. & LING, V. (1976). A surface glycoprotein modulating drug permeability in chinese hamster ovary cell mutants. *Biochim. Biophys. Acta*, **455**, 152–162.

KARTNER, N., EVERNDEN-PORELLE, D., BRADLEY, G. & LING, V. (1985). Detection of P-glycoprotein in multidrug-resistant cell lines by monoclonal antibodies. *Nature*, **316**, 820–823.

MANIATIS, T., FRITSCH, E.F. & SAMBROOK, J. (1982). *Molecular cloning, A Laboratory Manual.* Cold Spring Harbor, NY: Cold Spring Harbor Laboratory. 1982.

MOOS, M. & GALLWITZ, D. (1983). Structure of two human beta actin related processed genes one of which is located next to a simple repetitive sequence. *EMBO J.*, **2**, 727.

NOONAN, K.F., BECK, C., HOLZMAYER, T.A., CHIN, J.E., WUNDER, J.S., ANDRULIS, I.L., GAZDAR, A.F., WILLMAN, C.L., GRIFFITH, B., VON HOFF, D.D. & RONINSON, I.B. (1990). Quantitative analysis of MDR1 (multidrug resistance) gene expression in human tumors by polymerase chain reaction. *Proc. Natl Acad. Sci. USA*, **87**, 7160–7164.

NORMAND, E. & BLOCH, B. (1992). Protocoles pour la détection d'ARN-m par hybridation *in situ* par des sondes radioactives et biotinylées. In *Méthodes Pratiques.* Calas, A., Bloch, B., Fournié, J.G. & Trembleau, A. pp. 21–32. Paris, Société Francaise de microscopie électronique.

RONINSON, I.B., ABELSON, H.T., HOUSMAN, D.E., HOWELL, N. & VARSHAVSKY, A. (1984). Amplification of specific DNA sequences correlates with multidrug-resistance in chinese hamster cells. *Nature*, **309**, 626–628.

SAFA, A.R., KAPLAN STERN, R., CHOI, K., AGRESTI, M., TAMAI, I., MEHTA, N.D. & RONINSON, I.B. (1990). Molecular basis of preferential resistance to colchicine in multidrug-resistant human cells conferred by Gly-185-Val-185 substition in P-glycoprotein. *Proc. Natl Acad. Sci. USA*, **87**, 7225–7229.

SCHEPER, R.J., BULTE, J.W.M., BRAKKEE, J.G.P., QUAK, J.J., VAN DER SCHOOT, E., BALM, A.J.M., MEIJER, C.J.L.M., BROXTERMAN, H.J., KUIPER, C.M., LANKELMA, J. & PINEDO, H.M. (1988). Monoclonal antibody JSB1 detects a highly conserved epitope on the P-glycoprotein associated with multidrug-resistance. *Int. J. Cancer*, **42**, 389–394.

SCHLAIFER, D., LAURENT, G., CHITTAL, S., TSURUO, T., SUOUES, S., MULLER, C., CHARCOSSET, J.Y., ALARD, C., BROUSSET, P., MAZERROLLES, C. & DELSOL, G. (1990). Immunohistochemical detection of multidrug resistance associated P-glycoprotein in tumour and stromal cells of human cancers. *Br. J. Cancer*, **62**, 177–182.

SHEN, D.W., PASTAN, I. & GOTTESMAN, M.M. (1988). *In situ* hybridization analysis of acquisition and loss of the human multidrug-resistance gene. *Cancer Res.*, **48**, 4334–4339.

SUGAWARA, I., KATAOKA, I., MORISHITA, Y., HAMADA, H., TSURUO, T., ITOYAMA, S. & MORI, S. (1988). Tissue distribution of P-glycoprotein encoded by a multidrug-resistance gene as revealed by monoclonal antibody, MRK16. *Cancer Res.*, **48**, 1926.

TAWA, A., INOUE, M. & ISHIHARA, S. (1990). Increased expression of the multidrug-resistance gene in undifferentiated sarcoma. *Cancer*, **66**, 1980–1983.

THIEBAUT, F., TSURUO, T., HAMADA, H., GOTTESMAN, M.M., PASTAN, I. & WILLINGHAM, M.C. (1987). Cellular localization of the multidrug-resistance gene product P-glycoprotein in normal human tissues. *Proc. Natl Acad. Sci. USA*, **84**, 7735–7738.

TOFFOLI, G., FRUSTACI, S., TUMIOTTO, L., TALAMINI, R., GHERLINZONI, F., PICCI, P. & BOIOCCHI, M. (1992). Expression of MDR1 and GST- in human soft tissue sarcomas: Relation to drug resistance and biological aggressiveness. *Ann. Oncol.*, **3**, 63–69.

VAN DER VALK, P., VAN KALKEN, C.K., KETELAARS, H., BROXTERMAN, H.J., SCHEFFER, G., KUIPER, C.M., TSURUO, T., LANKELMA, J., MEIJER, C.J.L.M., PINEDO, H.M. & SCHEPER, R.J. (1990). *Ann. Oncol.*, **1**, 56–64.

WISHART, G.C., PLUMB, J.A., GOING, J.J., MCNICOL, A.M., MCARDLE, C.S., TSURUO, T. & KAYE, S.B. (1990). P-glycoprotein expression in primary breast cancer detected by immunocytochemistry with two monoclonal antibodies. *Br. J. Cancer*, **62**, 758–761.